# Antenatal and postnatal maternal mood symptoms and psychiatric disorders in pre-school children from the 2004 Pelotas Birth Cohort

**DOI:** 10.1016/j.jad.2014.04.033

**Published:** 2014-08-01

**Authors:** Iná S. Santos, Alicia Matijasevich, Aluísio J.D. Barros, Fernando C. Barros

**Affiliations:** a Programa de Pós-graduação em Epidemiologia, Universidade Federal de Pelotas, Rua Marechal Deodoro 1160, 3o piso, 96020-220 Pelotas, Brazil; b Programa de Pós-graduação em Saúde e Comportamento, Universidade católica de Pelotas, Pelotas, Rio Grande do Sul, Brazil

**Keywords:** DAWBA, Development and Well- Being Assessment, EPDS, Edinburgh Post-natal Depression Scale, LBW, low birth weight, LMP, last menstrual period, OR, odds ratio, MW, minimum wages, SRQ-20, self-reporting questionnaire, 95% CI, 95% confidence interval, Mood disorders, Self-reporting questionnaire, Development and Well-Being Assessment, Cohort study, Preschool children

## Abstract

**Background:**

Maternal mood symptoms have been associated with psychiatric disorders in children. This study aimed to assess critical periods when maternal symptoms would be more deleterious.

**Methods:**

Cohort of 4231 births followed-up in the city of Pelotas, Brazil. Mood symptoms during pregnancy were self-reported by mothers at perinatal interview; and at 3-months postpartum, mothers answered the Self-Reporting Questionnaire. Psychiatric disorders in 6-year-old children were evaluated through the Development and Well-Being Assessment instrument. Odds ratios with 95% confidence intervals (95% CI) were calculated by logistic regression.

**Results:**

Prevalence of mood symptoms in pregnancy was 24.6% (23.2–26.0%) and at three months postpartum 22.5% (21.1–23.9%). Prevalence of mental disorders in children was 13.3% (12.2–14.4%). After adjustment for confounders children of mothers with mood symptoms during pregnancy were 82% more likely of presenting psychiatric disorders than children of mothers that did not (1.82; 1.48–2.25); and the chance of having mental disorders among children whose mothers had positive SRQ-20 at three months postpartum was 87% greater than the observed among children whose mothers had it negative (1.87; 1.50–2.33).

**Limitations:**

Because maternal anxiety/depression may interfere with interpretation of the child behavior, child׳s mental health being obtained by interviewing the mother is a limitation of this study. Lack of information on other risk factors may have lead to residual confounding on the effect of maternal mood symptoms at three months postpartum.

**Conclusions:**

Children of mothers presenting mood symptoms during pregnancy and in the first months postpartum are more likely to present psychiatric disorders at 6 years of age.

## 1 Background

Parent׳s mental health, quality of the relationship between parents and children, level of their involvement and quality of care along with family socioeconomic conditions are important factors in the well-being and mental health of children (Waldfogel et al., 2010). The impact of these factors extends throughout life, becoming important preventable determinants of future mental disorders (Repetti and Seeman, 2002; Weich et al., 2009; Stewart-Brown and Schrader-Mcmillan, 2011).

Systematic reviews have shown that in high-income countries, about 10% of pregnant women and 13% of those who have given birth (Hendrick, 1998) experience some type of mental disorders, most commonly depression or anxiety (O’Hara and Swain, 1996). More recently, studies have shown that prevalence of non-psychotic common perinatal mental disorders was even higher in low- and lower-middle-income countries (15.6% antenatally and 19.8% postnatally) (Fisher et al., 2012). In these settings, risk factors included socioeconomic disadvantage, unintended pregnancy, younger age, being unmarried, lacking intimate partner empathy and support, having hostile in-laws, experiencing intimate partner violence, having insufficient emotional and practical support, in some settings, giving birth to a female, and having a history of mental health problems; whereas having more education, having a permanent job, belonging to the ethnic majority, and having a kind, trustworthy intimate partner were protective factors (Fisher et al., 2012).

The effect of affective disorders in pregnancy and of the presence of mental symptoms in the postpartum on the mental health of children has been the subject of numerous investigations, particularly in high-income countries. Most studies have shown that children of parents with a history of depression or anxiety are at an increased risk of developing psychiatric disorders (Lesesne et al., 2003; Lewinsohn et al., 2005), with internalizing syndromes being more common in these children than among those whose parents never had any mental disorder (Hammen and Pa, 2003; Lieb et al., 2002; Phillips et al., 2005; Weissman et al., 1997).

The identification of a critical point in the natural history of maternal mental disorders when interventions can achieve greater impact on the mental health of children would be desirable. Thus, the objective of this study was to investigate the effect of mood symptoms in pregnancy and at three months postpartum over mental disorders at six years of age in a population-based birth cohort in Pelotas, southern Brazil.

## 2 Methods

### 2.1 Participants

With approximately 340,000 inhabitants, Pelotas is a highly urbanized city where more than 90% of all deliveries take place in hospitals. From 1/1/2004 to 31/12/2004, all live births occurring in the city to mothers who were resident in the Pelotas urban area were enrolled in a cohort study (The 2004 Pelotas Birth Cohort). Births were assessed by daily visits to all five maternity hospitals. Mothers were interviewed soon after delivery by especially trained nutritionists with a pre-tested structured questionnaire. Detailed information was obtained about demographic, socio-economic, behavioral and biological characteristics, reproductive history, and health care utilization. Follow-ups were conducted at home at mean (SD) ages of 3.0 (0.1), 11.9 (0.2), 23.9 (0.4) and 49.5 (1.7) months and at a research clinic at 6.8 (0.3) years. The follow-up rates were between 90 and 96%. A detailed description of the methodology is given elsewhere (Santos et al., 2011).

### 2.2 Main exposures

Maternal mood during pregnancy was investigated at the perinatal interview by asking the question: *“During pregnancy, did you feel depressed or have any nervous condition*?*”* An affirmative answer was recorded as presence of mood symptoms during pregnancy. Maternal mental health at three months after parturition was evaluated through the application of a validated questionnaire, the Self-Reporting Questionnaire (SRQ-20) (Harpham et al., 2003; World Health Organization, 1994), developed by the World Health Organization to be used as a screening tool for psychiatric disorders. It consists of 20 items designed to identify symptoms that may be indicative of mental disorders. Each item is a question with possible yes/ no answers. The current version detects probable cases of anxiety/depression. A validation study in Brazil defined the cutoff≥8 to identify women in higher risk of anxiety/depression (sensitivity=83% and specificity=80%) (Mari and Williams, 1986).

### 2.3 Outcome

Children were assessed through maternal report using the Development and Well-Being Assessment (DAWBA) questionnaire (Goodman et al., 2000) validated in Brazil by Fleitlich-Bilyk & Goodman (Fleitlich-Bilyk and Goodman, 2004). The DAWBA consists of a structured questionnaire and open questions that allows to assess symptoms and generate psychiatric diagnoses among children aged 5 to 17 years according to the International Statistical Classification of Diseases and Related Health Problems 10th Revision (ICD-10) (World Health Organization, 1993) and the Diagnostic and Statistical Manual of Mental Disorders – Fourth Edition (DSM-IV) (American Psychiatric Association, 1994). Respondents initially completed the Strength and Difficulties Questionnaire (SDQ) (Goodman, 2001 before moving on to detailed interview sections covering a wide range of specific diagnoses. All DAWBA sections were explored: separation anxiety disorder, specific phobia, social phobia, generalized anxiety disorder, posttraumatic stress disorder, panic disorder and agoraphobia, obsessive-compulsive disorder, attention deficit hyperactivity disorder (ADHD), oppositional defiant disorder, conduct disorder, eating disorders, and tic disorders. In addition, five screening questions of a previous version of the DAWBA development section were utilized. DAWBA’s computer program proposes likely diagnoses. Two experienced clinical raters decided whether to accept or overturn the diagnoses in the light of all data and open questions. The outcome of interest was the presence of any of the above-mentioned diagnoses.

### 2.4 Covariates

Information about maternal and child׳s characteristics were gathered from the perinatal interview and from the three, twelve and 48-month follow-ups. Family income in the month prior to delivery was expressed as minimum wages (MW) (about US$ 80 in 2004) per month. Mother׳s formal education was categorized as 0–4, 5–8, 9–11 and ≥12 years of education. Maternal skin color was categorized as White and Black/Mixed according to the interviewer׳s observation. Women who were single, widowed, divorced or lived without a partner were classified as single mothers. Maternal age was defined as complete years at time of delivery. Parity was defined as the number of previous viable pregnancies resulting in a live birth or a late fetal death and categorized as 0, 1 and≥2. Mothers who smoked at least one cigarette per day in any trimester of pregnancy were categorized as smokers. Type of delivery (vaginal or cesarean section) and multiple births were recorded.

Birth weight was measured and recorded by the hospital staff with pediatric scales previously calibrated by the research team. Infants weighing less than 2500 g at birth were classified as low birth weight (LBW). Gestational age was calculated using the first day of the last normal menstrual period (LMP) or estimated by obstetric ultrasound obtained before 20 weeks of gestation when LMP was unreliable or not available. In the absence of both menstrual and ultrasound information, gestational age was estimated from physical and neurological assessment of the newborn Dubowitz’s et al. (1970). Preterm birth was defined as giving birth at<37 weeks of gestational age. Apgar׳s score at the fifth minute of life and type of hospital admission for the newborn (“staying with the mother” or “neonatal intensive care unit”) were recorded. Information on breastfeeding was collected at 3, 12, 24 and 48 months of age. Hospital admission during the first year of the child׳s life was defined as a readmission after being discharged in the post-delivery period.

### 2.5 Statistical analysis

Associations between maternal mood symptoms and the child׳s psychiatric disorders were analyzed by the chi-square test. Only potential confounders of the association between maternal mood symptoms and the child׳s psychiatric disorders at the 0.20 significance level were entered in the multivariate analyses, which were performed by logistic regression. Odds ratios (OR) with 95% confidence intervals (95% CI) were calculated. Duration of breastfeeding and hospitalization during the first year of the child׳s life were entered in the analyses as mediators of the association between maternal mood symptoms and child׳s psychiatric disorder. Low birth weight was considered a mediator in the association between maternal mood symptoms during pregnancy and child׳s psychiatric disorders, whereas it was considered a potential confounder for the association between positive SRQ-20 at the three months of age follow-up and child׳s psychiatric disorder. Positive SRQ-20 was considered a mediator in the association between symptoms during pregnancy and child׳s psychiatric disorders. All analyses were performed with Stata® version 11.0 software and the statistical significance level was set at *p*<0.05.

The Medical Ethics Committee of the Federal University of Pelotas, affiliated with the Brazilian Federal Medical Council, approved the study protocol and all follow-ups of the 2004 Pelotas Birth Cohort.

## 3 Results

A total of 3581 children with available information for psychiatric disorders at six years of age and for maternal mood symptoms were analyzed. Prevalence rates of mood symptoms in pregnancy and of positive SRQ-20 at the three months postpartum according to maternal characteristics are shown in Table 1. In the perinatal period interview, a total of 880 mothers (24.6%; 95% CI 23.2–26.0%) reported mood symptoms during pregnancy, which was inversely associated with household income and education. Higher prevalence of symptoms in pregnancy was also reported by mothers who lived without a partner and who were smokers during pregnancy. Prevalence of mood symptoms increased with age and parity.

At three months postpartum, the SRQ-20 was positive for 806 mothers (22.5%; 95% CI 21.1–23.9%), 42.0% of whom had reported mood symptoms in pregnancy at the perinatal interview. Just like for symptoms in pregnancy, the prevalence of symptoms at three months was inversely associated with household income and education (Table 1). Associations with marital status and smoking were similar to those observed with symptoms in pregnancy. As for age, the association was reversed: younger mothers had a higher prevalence (29.0%) than those aging more than 34 years (19.1%). Mothers living without a partner and those whose children were delivered vaginally had a higher prevalence of symptoms at three months. In Table 2, higher prevalence of symptoms was also observed among mothers of newborns with low birth weight (27.7% versus 22.0%), preterm (27.3% vs 21.8%) and hospitalized in the first year of life (28.7% vs. 20.4%).

The prevalence of psychiatric disorders diagnosed with the DSM-IV at six years of age in this study was 13.3% (95% CI 12.2–14.4%). The prevalence of psychiatric disorders was about two times higher among children of mothers who had reported mood symptoms in pregnancy (19.8% versus 11.1%) and at three months postpartum (21.7% versus 10.8%) (Table 3). Among the children of mothers who had reported symptoms in both occasions (pregnancy and at three months postpartum) the prevalence of psychiatric disorders was 25.1% (20.7–30.0%) versus the 9.5% (8.3–10.8%) found among those without such symptoms.

Among other maternal characteristics, higher prevalence of psychitric disorders were observed in children from families with lower incomes, children of mothers with less education, living without a partner and who smoked during pregnancy. Boys and those children hospitalized in the first year of life had a higher prevalence of psychiatric disorder at the age of six years old.

Table 4 presents the results of crude and adjusted analyses of the effect of maternal mood symptoms during pregnancy and at three months on the prevalence of mental disorders of children at six years of age. In the crude analysis, the odds of psychiatric disorder at six years of age was about twice as high among children whose mothers reported symptoms in pregnancy (OR=1.97; 95% CI 1.60–2.41) and 2.3 times higher among those whose mothers had positive SRQ-20 at three months postpartum (OR=2.29; 95% CI 1.86–2.81) in comparison, respectively, to children whose mothers did not report symptoms during pregnancy and who were not screening positive at three months.

Controlling for potential confounders did not change the association between symptoms in pregnancy and psychiatric disorders in children. The adjusted model for family income, maternal characteristics (education, marital status and smoking during pregnancy) showed 80% greater chance of psychiatric disorders among children of mothers with modd symptoms during pregnancy (OR=1.82; 95% CI 1.48 to 2.25) (Table 4). The inclusion of the mediators (low birth weight, duration of breastfeeding, hospitalization in the first year of life and positive SRQ-20 at three months) (models 4 and 5) decreased the value of the OR to 1.66, but the values of 95% CI (1.33 to 2.06) remained superimposed on the ones from the estimate adjusted for confounders.

After adjusting for confounders (including mood symptoms during pregnancy), the association between presence of mood symptoms at three months and the presence of psychiatric disorder in the child at six years of age was statistically significant (Table 4). The likelihood for psychiatric disorder was 87% higher among children whose mothers were SRQ-20 positive when compared to children of mothers who were SRQ-20 negative (OR=1.87; 95% CI 1:50–2:33). The inclusion of mediators (duration of breastfeeding and hospitalization in the first year of life) changed the OR to 1.78 with 95% confidence interval superimposed on the estimate adjusted for confounders.

## 4 Discussion

There were two main findings in this study. First, it was observed that in the studied population, mood symptoms in pregnancy and at three months postpartum presented long term effect on the mental health of children. Consistent with other studies (Center On The Developing Child At Harvard University, 2009; Avan et al., 2010; Verbeek et al., 2012; Weissman et al., 1997; Piche et al., 2011; Leis et al., 2013) it was found that six years after exposure to maternal emotional symptoms, children have increased chance to present psychiatric disorders.

Second, it was found that the strength of the association of maternal mental symptoms in pregnancy and at postpartum with psychiatric disorders in childhood was quite similar. Although the temporal proximity between the two measures of exposure could explain this finding it is also possible that those measures represent only two stages of the same disorder. For instance, postpartum maternal depression is considered by some as part of the natural history of depression, a *continuum* that begins before pregnancy and manifests throughout life, with critical recurrence in pre- and postnatal periods (Kessler et al., 2003). Although screening for maternal mental symptoms is not a universal practice, even in high-income countries, (Center On The Developing Child At Harvard University, 2009) an alternative explanation for the high prevalence in these periods would be the closer contact of the pregnant woman and later the mother in the first months of the baby׳s life with health services, which provides opportunities for screening and detection of mental problems.

In this study, the proportion of mothers who reported mood symptoms in pregnancy (about a quarter of the entire cohort) was very similar to the proportion that had positive SRQ-20 test in the third month postpartum. Also, about 40% of mothers that had reported mood symptoms in pregnancy showed positive screening at three months postpartum. Taking the total number of mothers of the cohort as the denominator, the prevalence of persistent mental symptoms was 12.6% (95% CI 11.6–13.8%).

Although mood symptoms may be episodic in pregnancy or postpartum, the present study points out the importance of investigating and handling maternal mood symptoms during prenatal care and in the months that follow birth. Particularly, there are numerous screening instruments for depression; many of them validated for the female population in postpartum which can be quickly and easily used in primary health care. There is also evidence that interventions provided by non-specialist mental health professionals, trained to support mothers in the perinatal period, are feasible and effective, thus improving maternal mental health, quality of mother-child relationship, and health, growth and development of children. (Rahman et al., 2013; Stewart-Brown and Schrader-Mcmillan, 2011)

### 4.1 Limitations of the study

Information about the child׳s mental health was obtained by interviewing the mother. It is possible that mothers who suffer from depression or anxiety may interpret the behavior of the child differently than a mother without mental disorders. A previous study showed that depressed mothers may overestimate problems in the behavior of their children (Van Der Toorn et al., 2010).

Moreover, as observed in other studies, children of mothers who experience depression are exposed to other risk factors such as environmental (low socioeconomic status), familiar (single mothers, violence and abuse by a partner, lack of social and emotional support) and maternal lifestyle (adolescent mothers with low education, drug abuse) (Jaffee et al., 2006; Brennan et al., 2002; Fisher et al., 2012) in addition to punishment with physical aggression as a method of discipline imposed on the child (Fatori et al., 2013), factors that may have a similar negative impact or even greater than maternal depression on child mental health (Barker et al., 2012). Not all of these factors were controlled in the present study; therefore part of the observed effect of maternal mood symptoms at three months may be due to residual confounding.

Also, the assessment of maternal mood during pregnancy using a single question (*“During pregnancy, did you feel depressed or have any nervous condition?”)* could be considered a limitation of the study. However, in previous analysis with mothers from the cohort aiming to identify longitudinal patterns of maternal depression between three months and 6 years postpartum, women in the “high-chronic” depression trajectory group reported the highest rates of positive answers to this question (unpublished data).

### 4.2 Strengths of the study

Among positive aspects, it should be pointed out that this study has the advantage of being population-based, not limited to specific groups of children and mothers with mental health problems, what favors its external validity. Because it is a cohort study, the temporality of the association between exposures and outcome is preserved. As the occurrence of exposures was investigated referring to the time of the interview or near its occurrence, reporting bias due to memory is reduced. Furthermore, the application of a tool for mental health diagnosis in childhood minimizes the risk of classification errors, increasing the validity of the definition of the outcome.

## 5 Conclusion

In this birth cohort study conducted in a middle-income country, prevalence of mothers with mood symptoms in prenancy and postpartum was high. Also high was the chance of children whose mothers presented mood symptoms in pregnancy and in the first months postpartum to present psychiatric disorders at six years of age. Based on the available evidence (Rahman et al., 2013), more attention should be paid to maternal affective symptoms during antenatal care as a way to prevent mental suffering of mothers and children.

## Role of funding source

The funding sources had no involvement in study design; collection, analysis, or interpretation of data; in the writing of the paper; or in the decision to submit the paper for publication.

## Conflict of interest

The Authors declare no conflict interests.

## Figures and Tables

**Table 1:** Description of the population, distribution of maternal mood symptoms in pregnancy and at 3 months postpartum, according to maternal characteristics (*n*=3581).

**Variables**	**Mood symptoms**				
	***n***	**Pregnancy*****n*****(%)**	***p***	**Three months postpartum*****n*****(%)**	***p***
Family income (minimum wages)			<0.001⁎		<0.001⁎
≤ 1	720	218(30.3)		226(31.4)	
1.1–3.0	1680	431(25.7)		415(24.7)	
3.1–6.0	805	171(21.2)		128(15.9)	
6.1–10.0	197	34(17.3)		24(12.2)	
>10.0	169	24(14.8)		11(6.5)	
Maternal education (years)			<0.001⁎		<0.001⁎
0–4	537	176(32.8)		192(35.8)	
5–8	1494	410(27.4)		406(27.2)	
9–11	1171	239(20.4)		181(15.5)	
≥ 12	344	51(14.8)		24(7.0)	
Maternal skin color			0.071⁎⁎		0.022⁎⁎
White	2627	625(23.8)		566(21.6)	
Black or mixed	954	255(26.7)		240(25.2)	
Marital status			0.001⁎⁎		0.001⁎⁎
With a partner	3010	708(23.5)		646(21.5)	
Alone	571	172(30.1)		160(28.0)	
Maternal age (years)			0.002⁎⁎		<0.001⁎
<20	683	161(23.6)		198(29.0)	
20–34	2406	567(23.6)		514(21.4)	
>34	491	152(31.0)		94(19.1)	
Parity			<0.001⁎		<0.001⁎
0	1424	293(20.6)		272(19.1)	
1	943	203(21.5)		182(19.3)	
≥ 2	1213	384(31.7)		352(29.0)	
Smoking in pregnancy			<0.001⁎⁎		<0.001⁎⁎
No	2611	579(22.2)		463(17.7)	
Yes	970	301(31.0)		343(35.4)	
Type of delivery			0.950⁎⁎		0.019⁎⁎
Vaginal	1950	480(24.6)		468(24.0)	
Cesarean	1631	400(24.5)		338(20.7)	
All	3581	880(24.6)		806(22.5)	

⁎ Chi-squared test for trend.

⁎⁎ Chi-squared test.

**Table 2:** Description of the population and distribution of maternal mood symptoms at 3 months postpartum, according to children׳s characteristics (*n*=3581).

Variables	*n*	Mood symptoms at 3 months postpartum*n* (%)	*p*

Multiple birth			0.516
No	3511	788(22.4)	
Yes	70	18(25.7)	
Gender			0.816
Male	1862	422(22.7)	
Female	1719	384(22.3)	
Birth weight<2500 g			0.021
No	3262	718(22.0)	
Yes	318	88(27.7)	
Gestational age<37 weeks			0.007
No	3089	672(21.8)	
Yes	488	133(27.3)	
Apgar<7 at the fifth minute of life			0.274
No	3502	784(22.4)	
Yes	60	17(28.3)	
NICU⁎ at birth			0.146
No	3257	721(22.1)	
Yes	315	81(25.7)	
Hospitalization in the first year of life			<0.001
No	2830	578(20.4)	
Yes	649	186(28.7)	
Duration of breastfeeding (months)			0.077
Never breastfed	95	28(29.5)	
<12	2111	483(22.9)	
12.0–23.9	593	113(19.1)	
≥24.0	772	172(22.3)	
All	3581	806(22.5)	

⁎ NICU: neonatal intensive care unit.

**Table 3:** Distribution of psychiatric disorders assessed through the Development and Well-Being Assessment (DAWBA) instrument at 6 years of age, according to maternal and child׳s characteristics.

Variables	**Psychiatric disorders*****n*****(%)**	***p***
Family income (minimum wages)		<0.001
≤1	108(15.0)	
1.1–3.0	247(14.7)	
3.1–6.0	94(11.7)	
6.1–10.0	17(8.6)	
>10.0	7(4.1)	
Maternal education (years)		0.002
0–4	74(13.8)	
5–8	235(15.7)	
9–11	132(11.3)	
≥12	34(9.9)	
Maternal skin color		0.619
White	344(13.1)	
Black or mixed	131(13.7)	
Marital status		0.010
With a partner	380(12.6)	
Alone	95(16.6)	
Maternal age (years)		0.261
<20	96(14.1)	
20–34	325(13.5)	
>34	54(11.0)	
Parity		0.216
0	190(13.3)	
1	111(11.8)	
≥2	174(14.3)	
Smoking in pregnancy		<0.001
No	309(11.8)	
Yes	166(17.1)	
Mood symptoms in pregnancy		<0.001
No	301(11.1)	
Yes	174(19.8)	
Type of delivery		0.306
Vaginal	269(13.8)	
Cesarean	206(12.6)	
Gender		0.006
Male	275(14.8)	
Female	200(11.6)	
Birth weight<2500 g		0.115
No	441(13.5)	
Yes	33(10.4)	
Gestational age<37 weeks		0.924
No	410(13.3)	
Yes	64(13.1)	
Apgar<7 at the fifth minute of life		0.116
No	458(13.1)	
Yes	12(20.0)	
NICU⁎ at birth		0.260
No	423(13.0)	
Yes	48(15.2)	
Mood symptoms at 3 months postpartum		<0.001
No	300(10.8)	
Yes	175(21.7)	
Hospitalization in the first year of life		0.005
No	350(12.4)	
Yes	107(16.5)	
Duration of breastfeeding (months)		0.274
Never breastfed	17(17.9)	
<12	266(12.6)	
12.0–23.9	88(14.8)	
≥24.0	101(13.1)	
All	475(13.3)	

⁎ NICU: neonatal intensive care unit.

**Table 4:** Crude and adjusted analysis of the association between maternal mood symptoms during pregnancy and at 3 months postpartum with children psychiatric disorders at 6 years of age.

	**Odds ratio of psychiatric disorders at 6 years of age (95% CI)**	***p***
**Association between maternal mood symptoms in pregnancy and psychiatric disorders at 6 years of age**		
Model 1: Crude	1.97(1.60; 2.41)	<0.001
Model 2: Model 1+family income, maternal education and marital status	1.85(1.50; 2.28)	<0.001
Model 3: Model 2+maternal smoking in pregnancy	1.82(1.48; 2.25)	<0.001
Model 4: Model 3+low birth weight (mediator)	1.83(1.48; 2.25)	<0.001
Model 5: Model 4+duration of breastfeeding, hospitalization in the first year of life and maternal SRQ (mediators)	1.66(1.33; 2.06)	<0.001

**Association between positive SRQ at 3 months postpartum and psychiatric disorders at 6 years of age**		
Model 1: Crude	2.29(1.86; 2.81)	<0.001
Model 2: Model 1+family income, maternal education and marital status	2.13(1.72; 2.63)	<0.001
Model 3: Model 2+maternal smoking and depression in pregnancy	1.85(1.48; 2.31)	<0.001
Model 4: Model 3+low birth weight	1.87(1.50; 2.33)	<0.001
Model 5: Model 4+duration of breastfeeding and hospitalization in the first year of life (mediators)	1.78(1.42; 2.24)	<0.001
